# Direct Growth of Platinum Monosulfide Nanoparticles on MXene via Single‐Source Precursor for Enhanced Hydrogen Evolution Reaction

**DOI:** 10.1002/smsc.202500407

**Published:** 2025-09-15

**Authors:** Younghee Park, Chanwon Park, Sunyoung Shin, Da Som Song, Myung Hyun Kang, Chang Gyoun Kim, Yun Chan Kang, Sung Myung, Jongsun Lim

**Affiliations:** ^1^ Thin Film Materials Research Center Korea Research Institute of Chemical Technology Daejeon 34114 Republic of Korea; ^2^ Department of Materials Science and Engineering Korea University Anam‐dong, Seongbuk‐gu Seoul 136‐713 Republic of Korea

**Keywords:** electrocatalysis, hydrogen evolution reaction, MXene, PtS, single‐source precursor

## Abstract

2D Ti_3_C_2_T_x_ MXene offers high electrical conductivity and a large surface area, making it attractive for electrocatalysis. However, its intrinsic hydrogen evolution reaction (HER) activity remains poor due to the lack of active catalytic sites. To activate the otherwise inert surface, platinum monosulfide (PtS) nanoparticles are synthesized directly on Ti_3_C_2_T_x_ nanosheets via thermal decomposition of a single‐source precursor, Pt(dmampS)_2_, in a solution‐based process. This direct growth strategy enables uniform dispersion of PtS nanoparticles and intimate interfacial contact with the MXene surface, without the need for binders or surfactants. The resulting PtS/Ti_3_C_2_T_x_ heterostructure exhibits significantly enhanced HER performance, achieving a low overpotential of −104 mV at a current density of −10 mA cm^−2^ and a Tafel slope of 48.3 mV dec^−1^.

## Graphical abstract

Direct growth of platinum monosulfide (PtS) nanoparticles on Ti_3_C_2_T_x_ MXene via a single‐source precursor creates robust interfacial coupling and efficient charge transfer. The PtS/MXene heterostructure demonstrates superior hydrogen evolution activity and remarkable durability compared to standalone PtS or pristine Ti_3_C_2_T_x_, highlighting the synergistic effect and scalable potential of Pt‐based chalcogenide/MXene electrocatalysts.

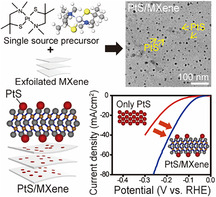

## 1 Introduction

2D materials have attracted significant attention in electrocatalysis owing to their high surface area, tunable electronic structures, and excellent mechanical and chemical stability. These features enable efficient charge transport and provide effective interfaces for surface catalytic reactions, including the hydrogen evolution reaction (HER). MXenes, a family of 2D transition metal carbides and nitrides with the general formula M_n+1_X_n_T_x_ (where M is a transition metal, X is carbon or nitrogen, and T represents surface terminations such as fluorine, chlorine, oxygen, or hydroxyl groups), offer diverse compositions and tunable surface functionalities.^[^ ^1^, ^2^, ^3^, ^4^, ^5^, ^6^ ^]^ Among them, Ti_3_C_2_T_x_ MXene has been extensively studied due to its high electrical conductivity, hydrophilic surface, and controllable terminations, which facilitate electron transport and interfacial engineering for electrocatalysis.^[^ ^7^, ^8^, ^9^, ^10^ ^]^ However, despite these advantages, pristine Ti_3_C_2_T_x_ exhibits negligible intrinsic HER activity due to the lack of active catalytic sites. While its surface hosts various terminal groups, they do not provide optimal hydrogen adsorption energy (Δ*G*_H_), essential for efficient HER.^[^ ^11^, ^12^, ^13^, ^14^, ^15^ ^]^ Moreover, the Ti metal centers bind hydrogen weakly, resulting in insufficient active sites for hydrogen adsorption and desorption. Consequently, pristine Ti_3_C_2_T_x_ displays high overpotentials and low current densities during HER, indicating poor catalytic performance despite its excellent conductivity and surface area.

To address this limitation, recent studies have explored hybridizing Ti_3_C_2_T_x_ with catalytically active materials to activate its otherwise inert surface.^[^ ^8^, ^15^, ^16^, ^17^, ^18^ ^]^ Among them, Pt‐based nanostructures have demonstrated outstanding HER performance due to their near‐optimal hydrogen binding energy, which facilitates both adsorption and desorption of hydrogen intermediates.^[^ ^19^, ^20^, ^21^, ^22^, ^23^, ^24^ ^]^ However, the high cost and scarcity of Pt, along with its weak adhesion to conventional supports and poor long‐term durability under electrochemical conditions, hinder its practical application.^[^ ^25^, ^26^, ^27^ ^]^

Platinum monosulfide (PtS) is considered a promising alternative to conventional Pt catalysts, offering reduced Pt usage while retaining excellent catalytic activity. Here, we employ a single‐source precursor approach using Pt(dmampS)_2_ to directly grow PtS nanoparticles on exfoliated Ti_3_C_2_T_x_ MXene, forming a well‐integrated PtS/Ti_3_C_2_T_x_ heterostructure. This direct growth method ensures uniform nanoparticle dispersion and strong interfacial coupling without requiring additional binders or surfactants. The resulting heterostructure introduces abundant catalytic sites on the otherwise inert MXene surface, significantly enhancing HER activity with reduced Pt content. Electrochemical results confirm that the precursor‐driven synthesis produces uniform, stable nanostructures with minimized aggregation and improved catalytic durability.

## 2 Results and Discussions

### 2.1 Morphology and Structural Analysis of PtS/Ti_3_C_2_T_x_ Heterostructure

**Figure 1:**
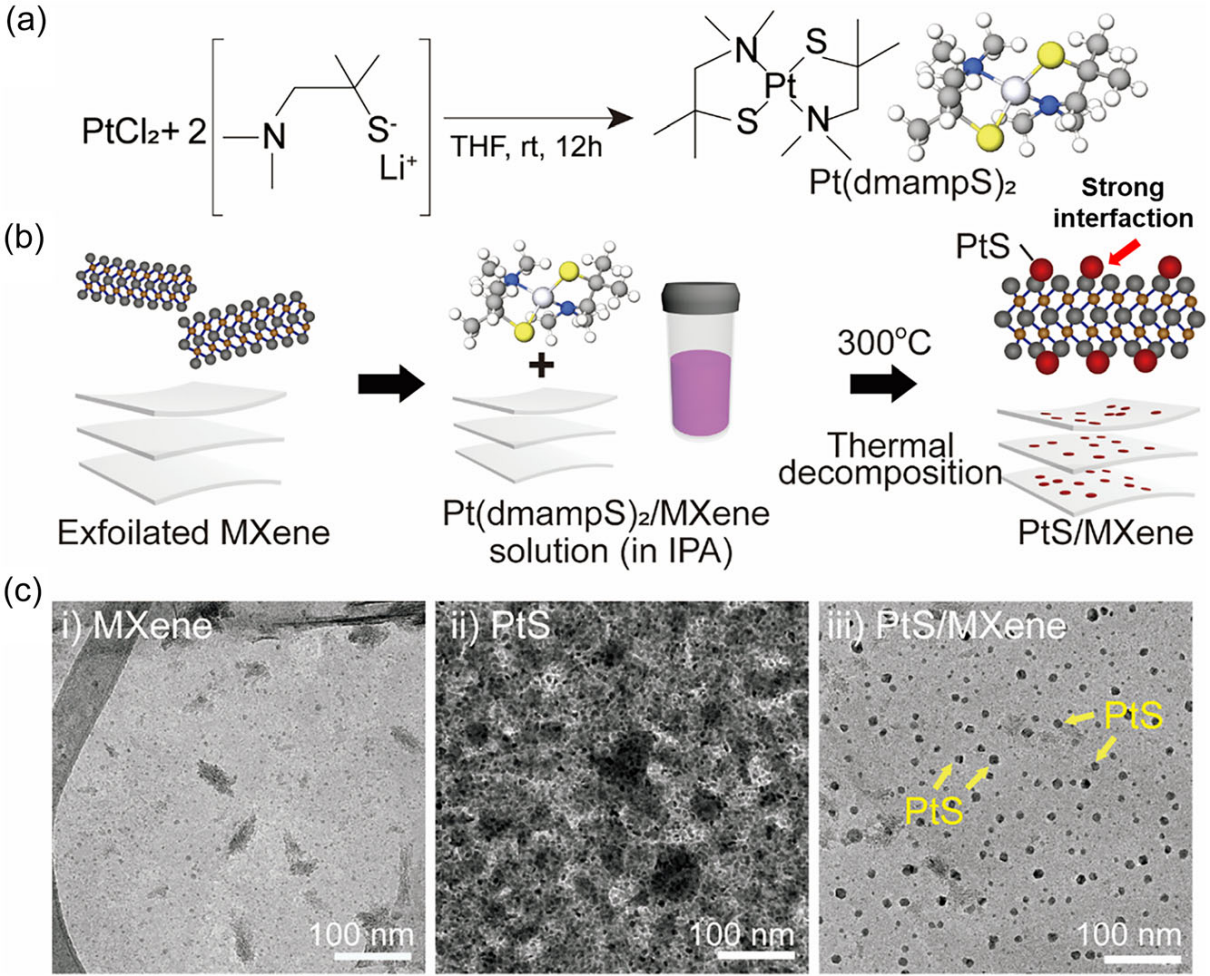
Synthesis and structural characterization of PtS‐decorated Ti_3_C_2_T_x_ MXene heterostructure. a) Schematic illustration of synthesis process of Pt(dmampS)_2_, b) schematic of fabrication PtS/Ti_3_C_2_T_x_ catalyst from exfoliated Ti_3_C_2_T_x_ MXene with Pt(dmampS)_2_, c) HR‐TEM images of (i) Ti_3_C_2_T_x_ MXene, (ii) PtS, and (iii) PtS/Ti_3_C_2_T_x_ MXene.

The single‐source precursor Pt(dmampS)_2_ was synthesized and utilized for the direct growth of PtS nanoparticles, as illustrated in **Figure** **1**a (see details in Experimental Section 4.1). The molecular structure of Pt(dmampS)_2_ consists of a Pt(II) center coordinated by two bidentate amino‐thiolate ligands (Figure 1a). This monomeric configuration ensures both molecular volatility and excellent solution processability, while also providing favorable sulfur‐based anchoring interactions with the Ti_3_C_2_T_x_ MXene surface. Thermogravimetric analysis (TGA) further confirmed the suitability of Pt(dmampS)_2_ as a single‐source precursor, exhibiting rapid decomposition at relatively low temperatures below 240 °C (Figure S1, Supporting Information). These structural and thermal characteristics enable efficient, low‐temperature conversion into PtS nanoparticles without the need for additional reducing agents or surfactants. To confirm the thermal stability of Ti_3_C_2_T_x_ under these annealing conditions, we conducted X‐ray diffraction (XRD) and Raman spectroscopy analyses (Figure S2, Supporting Information). XRD results showed slight restacking of Ti_3_C_2_T_x_ layers after annealing at 300 °C, as evidenced by a characteristic peak shift from 7° to 8°. Nevertheless, Raman spectra confirmed that the intrinsic layered structure of Ti_3_C_2_T_x_ remained intact without signs of phase degradation or oxidation.^[^ ^28^, ^29^ ^]^ To synthesize PtS/Ti_3_C_2_T_x_ heterostructures, exfoliated Ti_3_C_2_T_x_ sheets were mixed with the Pt(dmampS)_2_ precursor in solution, drop‐cast onto a carbon substrate, and thermally annealed at 300 °C under an inert atmosphere (Figure 1b). This direct growth process facilitates uniform nanoparticle distribution across the MXene surface without requiring surfactants or binders. For structural and morphological characterization, pristine Ti_3_C_2_T_x,_ pristine PtS nanoparticles, and PtS‐decorated Ti_3_C_2_T_x_ heterostructures were prepared and examined by high‐resolution transmission electron microscopy (HR‐TEM) (Figure 1c). As shown in Figure 1c–i, exfoliated Ti_3_C_2_T_x_ exhibits a continuous, well‐separated layered structure. In addition, atomic force microscopy (AFM) analysis revealed a thickness of ≈3.5 nm, confirming the formation of a few‐layer MXene sheet (Figure S3, Supporting Information). In contrast, pristine PtS nanoparticles synthesized without the MXene support tend to aggregate severely, as shown in Figure 1c‐ii. By comparison, PtS nanoparticles in the PtS/Ti_3_C_2_T_x_ heterostructure are uniformly distributed across the MXene surface, indicating successful nanoparticle anchoring and suppressed aggregation (Figure 1c‐iii).

**Figure 2:**
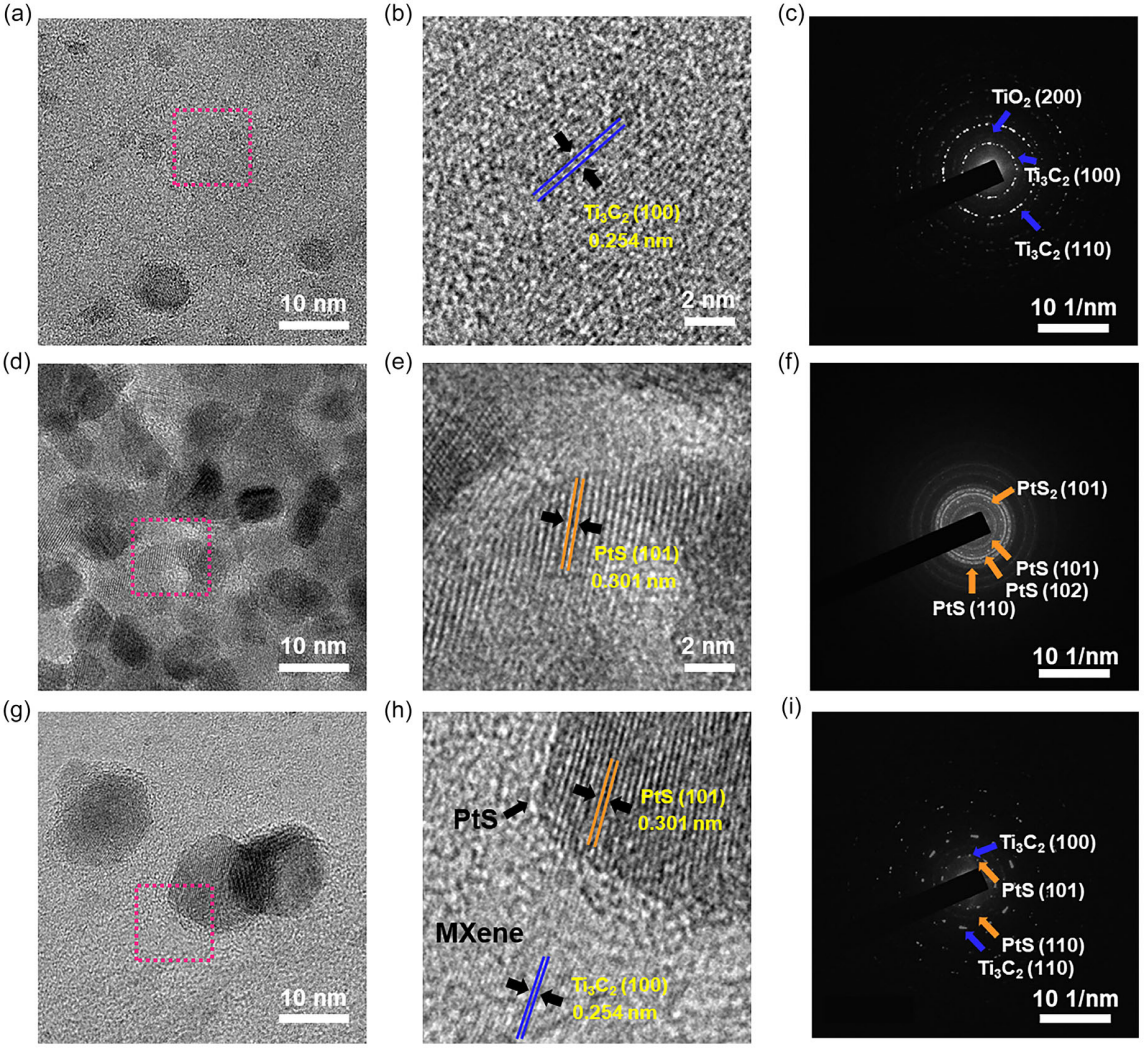
TEM and SAED analysis of Ti_3_C_2_T_x,_ PtS nanoparticles, and PtS/Ti_3_C_2_T_x_ heterostructure. a) HR‐TEM images of Ti_3_C_2_T_x_. b) lattice fringe of Ti_3_C_2_T_x_ MXene measured from the area marked by the red dotted square in (a). c) SAED pattern obtained from Ti_3_C_2_T_x_ MXene. d) HR‐TEM images of aggregated PtS nanoparticles. e) lattice fringe of PtS nanoparticle measured from the area marked by the red dotted square in (d). f) SAED pattern obtained from aggregated PtS nanoparticles. g) HR‐TEM images of PtS/Ti_3_C_2_T_x_. h) lattice fringe of PtS/Ti_3_C_2_T_x_ measured from the area marked by the red dotted square in (g). i) SAED pattern obtained from PtS/Ti_3_C_2_T_x_.

To further investigate the lattice structure and interfacial contact, HR‐TEM and selected area electron diffraction (SAED) analyses were performed on exfoliated Ti_3_C_2_T_x_ (**Figure** **2**a–c), pristine PtS nanoparticles (Figure 2d–f), and PtS‐decorated Ti_3_C_2_T_x_ heterostructures (Figure 2g–i). Exfoliated Ti_3_C_2_T_x_ displays a layered morphology with clearly resolved lattice fringes corresponding to the (100) plane of Ti_3_C_2_ (0.254 nm), as shown in Figure 2a and b.^[^ ^30^ ^]^ The corresponding SAED pattern (Figure 2c) exhibits distinct diffraction spots assigned to the (100) and (110) planes of Ti_3_C_2_T_x_, confirming the high crystallinity of the exfoliated layers. A weak diffraction ring corresponding to the (200) plane of TiO_2_ is also observed, suggesting minor surface oxidation during the exfoliation process. In contrast, PtS nanoparticles synthesized without the Ti_3_C_2_T_x_ substrate exhibit aggregated polycrystalline domains, with observed lattice spacings such as 0.301 nm matching the (101) plane of tetragonal PtS (Figure 2d and e). The SAED pattern (Figure 2f) reveals ring‐like features characteristic of polycrystalline PtS nanoparticles.^[^ ^31^, ^32^ ^]^ These diffraction rings can be indexed to the (101) and (102) planes of tetragonal PtS (ICSD‐654 379, space group: P4_2_/mmc, *a* = *b* = 3.47 Å, *c* = 6.11 Å, *α = β = γ* = 90°), along with a weak signal from the (101) plane of PtS_2_, indicating partial coexistence or surface oxidation of PtS under the synthesis conditions. For the PtS/ Ti_3_C_2_T_x_ heterostructure (Figure 2g–i), PtS nanoparticles are uniformly distributed across the MXene surface, maintaining distinct lattice fringes. The interfacial region shows no visible voids, and the nanoparticles appear firmly anchored to the Ti_3_C_2_T_x_ substrate (Figure S4, Supporting Information). Slight lattice distortion near the interface suggests strong interfacial coupling between PtS and Ti_3_C_2_T_x_. The SAED pattern (Figure 2i) exhibits sharp, discrete spots originating from both the PtS nanoparticles and the Ti_3_C_2_T_x_ layers. The diffraction spots correspond to the (100) and (110) planes of Ti_3_C_2_T_x_ and the (101) and (110) planes of tetragonal PtS, confirming the formation of a structurally coherent heterointerface. In addition, TEM‐EDS spot analysis (Figure S5, Supporting Information) further confirmed that Ti peaks dominated in the Ti_3_C_2_T_x_ region, while both Pt and Ti signals were detected in the PtS/Ti_3_C_2_T_x_ region, demonstrating the coexistence of PtS and Ti_3_C_2_T_x_ in the heterostructure. These results demonstrate the successful construction of an integrated PtS/Ti_3_C_2_T_x_ heterostructure with strong interfacial contact.

### 2.2 Surface Chemical States and Evidence of Strong Interfacial Coupling

**Figure 3:**
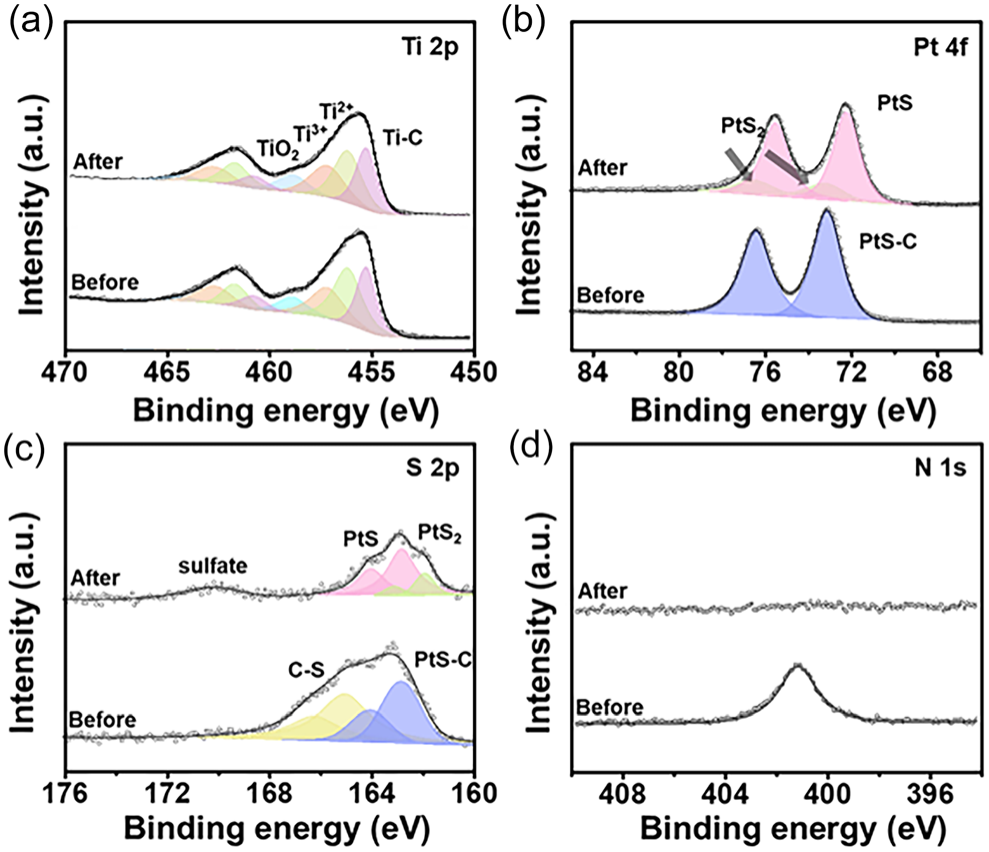
XPS spectra of a) Ti 2p, b) Pt 4f, c) S 2p, and d) N 1s for PtS/Ti_3_C_2_T_x_ before and after annealing.

To investigate the surface composition and chemical states of the PtS/Ti_3_C_2_T_x_ heterostructure, X‐ray photoelectron spectroscopy (XPS) analysis was performed on the Pt(dmampS)_2_/Ti_3_C_2_T_x_ mixture before and after thermal annealing at 300 °C. The preannealed sample was prepared by mixing exfoliated Ti_3_C_2_T_x_ with the Pt(dmampS)_2_ precursor in solution, while the postannealed sample corresponds to the PtS/Ti_3_C_2_T_x_ heterostructure obtained via thermal decomposition. The XPS survey spectra of both samples are shown in Figure S6a, Supporting Information, where elements including C, Ti, O, F, Cl, Pt, and S are detected. **Figure** **3**a presents the Ti 2p spectra of the composite before and after annealing. Both spectra exhibit four distinct peaks, corresponding to Ti—C bonding, Ti^2+^, Ti^3+^, and TiO_2_ species, indicating the coexistence of carbide and oxidized surface states in the Ti_3_C_2_T_x_ MXene. The deconvoluted peaks appear at binding energies of 455.3 (460.8 eV), 456.2 (461.7 eV), 457.2 (462.7 eV), and 458.9 eV (464.4 eV), assigned to Ti—C, Ti^2+^, Ti^3+^, and TiO_2_, respectively.^[^ ^33^ ^]^ The negligible change in these features after annealing confirms that the MXene framework remains structurally and chemically stable under the applied thermal decomposition conditions. The successful conversion of Pt(dmampS)_2_ to PtS was verified by XPS analysis of the Pt 4f and S 2p regions (Figure 3b and c). In the preannealed sample, the Pt 4f peaks appear at 72.9 (76.3 eV) and 73.5 eV (76.8 eV), attributed to Pt^2+^ species coordinated with thiolate and amino ligands in the Pt(dmampS)_2_ precursor. After annealing, these peaks shift to 72.2 eV (75.5 eV), characteristic of Pt^2+^ in PtS, confirming the successful formation of PtS nanoparticles on the Ti_3_C_2_T_x_ surface.^[^ ^34^ ^]^ Similar trends are observed in the S 2p spectra. Prior to annealing, the S 2p region displays broad and asymmetric peaks between 160 and 172 eV, attributed to sulfur species coordinated to the Pt center in the precursor. After annealing, two well‐defined peaks emerge at 162.8 (164.0 eV) and 161.9 eV (163.1 eV), corresponding to PtS and PtS_2_, respectively. This transformation confirms the complete decomposition of the organosulfur ligands and the formation of sulfide phases. Additionally, the N 1s signal observed at 401.1 eV in the preannealed sample, originating from the amino ligands of Pt(dmampS)_2_, completely disappears after annealing, indicating the full removal of nitrogen‐containing species. This result is further corroborated by changes in the C 1s spectra (Figure S6b, Supporting Information).

### 2.3 Electrochemical Assessment of HER Activity and Stability in PtS/Ti_3_C_2_T_x_

**Figure 4:**
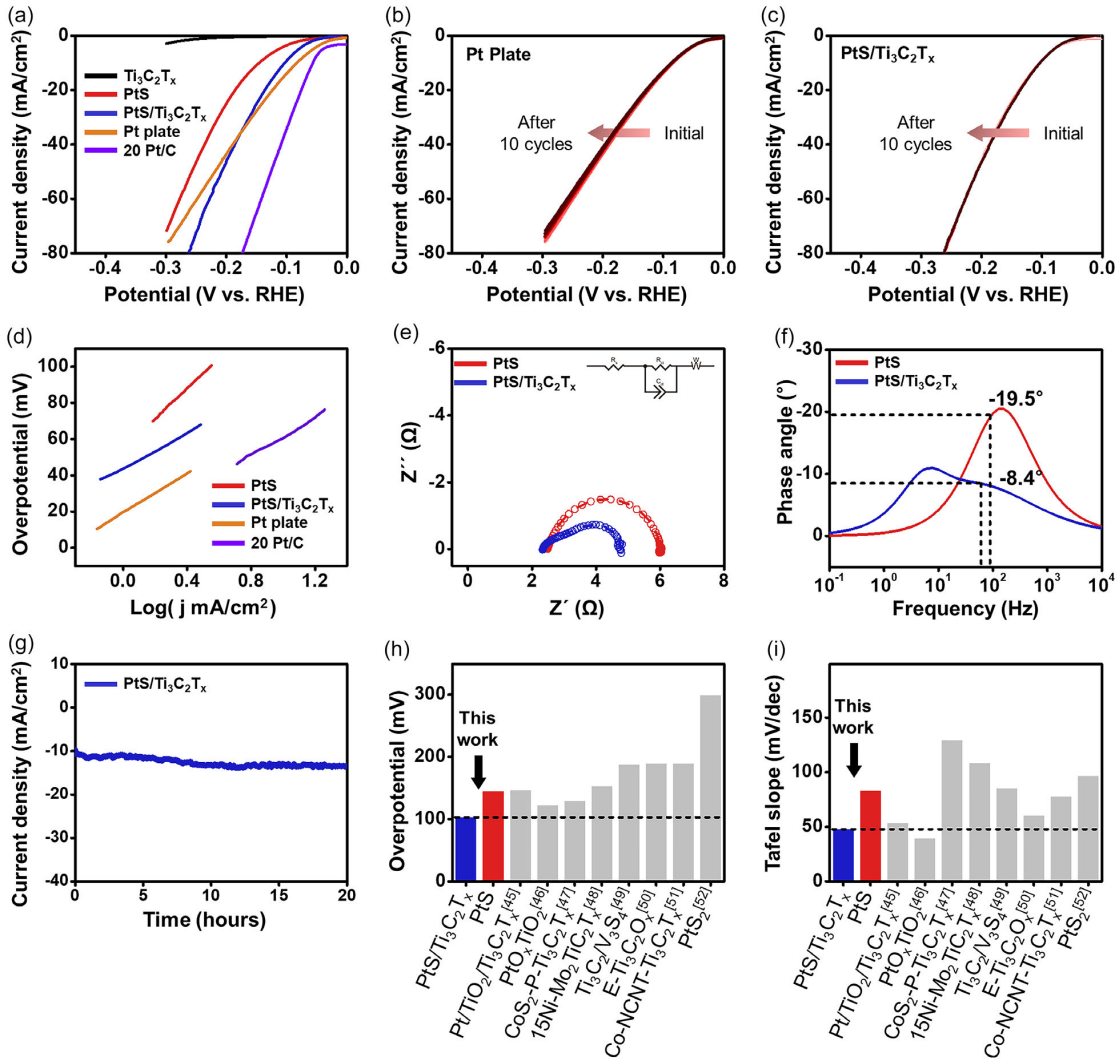
Electrocatalytic performance and stability evaluation of PtS/Ti_3_C_2_T_x_ heterostructure for hydrogen evolution reaction. a) LSV curves of HER catalysts, Ti_3_C_2_T_x_ (black), PtS (red), PtS/Ti_3_C_2_T_x_ (blue), Pt plate (orange), and 20 Pt/C (violet), b) cycle repeatability of Pt plate and c) PtS/Ti_3_C_2_T_x_ , d) Tafel slopes from LSV curves, e) Nyquist plot of PtS (red) and PtS/Ti_3_C_2_T_x_ (blue) fitted using the equivalent circuit inset figure, f) phase angle of PtS (red) and PtS/Ti_3_C_2_T_x_ (blue), g) chronoamperometry curve of PtS/Ti_3_C_2_T_x_ at overpotential of −10 mA cm^−2^ current density, summary of HER catalyst performance, h) overpotential and i) Tafel slope.

The electrochemical performance of the prepared HER catalysts was evaluated using linear sweep voltammetry (LSV) in a 0.5 M H_2_SO_4_ electrolyte, within the potential window of 0 to −0.3 V versus the reversible hydrogen electrode (RHE) at a scan rate of 5 mV s^−1^. A commercial Pt plate and 20 Pt/C were measured under identical conditions for comparison. **Figure** **4**a shows the LSV curves for annealed Ti_3_C_2_T_x_ (black), PtS nanoparticles (red), PtS/Ti_3_C_2_T_x_ heterostructure (blue), the commercial Pt plate (orange), and 20 Pt/C (violet). As expected, Ti_3_C_2_T_x_ displays negligible catalytic activity due to its inert basal plane, despite its excellent conductivity and hydrophilicity. PtS exhibits an overpotential of −146 mV at a current density of –10 mA cm^−2^, while the PtS/Ti_3_C_2_T_x_ achieves a significantly reduced overpotential of −104 mV under the same conditions. The enhanced performance of the heterostructure is attributed to the synergistic effect between Ti_3_C_2_T_x_, which facilitates efficient charge transport and interfacial wettability, and PtS, which serves as the primary catalytic site. The overpotential of the commercial Pt plate and 20 Pt/C shows −83.8 and ‐57.1 mV, respectively. The cycle stability of PtS/Ti_3_C_2_T_x_ was evaluated by LSV over 10 consecutive cycles, compared to the commercial Pt plate (Figure 4b,c). During HER, hydrogen bubble accumulation on the catalyst surface can reduce active site availability. However, hydrophilic surface groups on HER catalyst facilitate water intercalation at the electrode–electrolyte interface, enhancing bubble detachment and maintaining HER performance.^[^ ^35^, ^36^, ^37^ ^]^ The overpotentials of the commercial Pt plate are continuously decreased slightly due to H_2_ bubbles on the Pt surface. By contrast, PtS/Ti_3_C_2_T_x_ exhibits almost the same overpotential values at −10 mA cm^−2^ current density because of the intercalation of the H_2_O molecules between the electrode surface and electrolyte. Moreover, in the high current density regions, hydrogen bubble accumulation can block active sites, diminishing HER performance. So, a current density of −50 mA cm^−2^, PtS/Ti_3_C_2_T_x_ exhibits an overpotential of −208 mV, which is slightly lower than that of the commercial Pt plate (−219 mV) due to elimination of H_2_ bubbles on the PtS/Ti_3_C_2_T_x_ surface. Overall, the integration of Ti_3_C_2_T_x_ into PtS significantly enhances HER performance, particularly under high current density conditions (i.e., >−40 mA cm^−2^). The catalytic activity was further assessed by Tafel slope analysis based on the Tafel equation (*η = b log j  +  a,* where *η* is the overpotential, *b* is the Tafel slope, and *j* is the current density).^[^ ^38^, ^39^ ^]^ As summarized in Figure 4d, the Tafel slope of PtS alone is 83.5 mV dec^−1^, while that of PtS/Ti_3_C_2_T_x_ is reduced to 48.3 mV dec^−1^. This substantial decrease is attributed to the improved charge transfer and increased density of active sites resulting from the heterostructure formation. Remarkably, the Tafel slope of PtS/Ti_3_C_2_T_x_ is also lower than that of the commercial Pt plate (54.1 mV dec^−1^) and 20 Pt/C (51.8 mV dec^−1^), highlighting the advantageous interfacial properties of the composite catalyst. The intrinsic activity of the PtS/Ti_3_C_2_T_x_ is evaluated using turnover frequency (TOF), and the calculated TOF at −104 mV overpotential is 4.43 s^−1^, which is a higher value of 20 Pt/C (3.50 s^−1^).

Electrochemical impedance spectroscopy (EIS) was conducted under a bias potential of –0.2 V versus RHE to examine charge transfer resistance during HER. Nyquist plots and fitted data for PtS and PtS/Ti_3_C_2_T_x_ are shown in Figure 4e, with the equivalent circuit illustrated in the inset; results for Ti_3_C_2_T_x_ are provided in Figure S7a, Supporting Information. The equivalent circuit comprises three elements: series resistance (*R*_*s*_) representing the total resistance of the catalyst and electrolyte; diffusion resistance (*W*_*s*_) related to proton transport; and charge transfer resistance (*R*_*ct*_) coupled with a constant phase element (*C*_*dl*_) reflecting interfacial processes.^[^ ^40^, ^41^, ^42^ ^]^ Annealed Ti_3_C_2_T_x_ exhibited the lowest *R*_*s*_ (2.26 Ω) due to its intrinsic high conductivity. Incorporating Ti_3_C_2_T_x_ into the PtS/Ti_3_C_2_T_x_ heterostructure reduced *R*_*s*_ from 2.48 (PtS) to 2.30 Ω, confirming improved electrode conductivity. The *R*_*ct*_ value of PtS/ Ti_3_C_2_T_x_ (0.97 Ω) represents a 2.9‐fold improvement over PtS (2.83 Ω) and a remarkable 124.9‐fold enhancement compared to annealed Ti_3_C_2_T_x_ (105 Ω), highlighting the synergistic effect of the heterostructure on charge transfer. The relaxation frequency of PtS/Ti_3_C_2_T_x_ (68.6 Hz) is lower than that of PtS (96.5 Hz), attributed to partial restacking of 2D materials, which increases proton diffusion resistance (Ws = 1.49 Ω for PtS/ Ti_3_C_2_T_x_ vs. 0.70 Ω for PtS). In comparison, annealed Ti_3_C_2_T_x_ shows a significantly lower relaxation frequency (0.11 Hz) and high diffusion resistance (15.9 Ω) due to its poor catalytic activity. The Bode plots in Figure 4f and Figure S7b, Supporting Information illustrate the phase angle as a function of frequency. A higher phase angle at the relaxation frequency implies sluggish interfacial kinetics.^[^ ^42^, ^43^, ^44^ ^]^ Among the samples, annealed Ti_3_C_2_T_x_ exhibits the highest phase angle (−45°), followed by PtS (−19.5°), while PtS/Ti_3_C_2_T_x_ shows the lowest value (−8.4°), further confirming that heterostructure formation effectively accelerates the charge transfer process. Long‐term durability was further assessed by accelerated durability test (ADT) consisting of 1,000 continuous cyclic voltammetry (CV) cycles between –40 mV and 0 V versus RHE at a scan rate of 100 mV s^−1^ in 0.5 M H_2_SO_4_, and chronoamperometry (CA) measurement with constant bias of 10 mA cm^−2^ overpotential. As shown in Figure S8, Supporting Information, PtS/Ti_3_C_2_T_x_ retained ≈92% of its initial current density after 1,000 cycles at –0.1 V versus RHE, outperforming previously reported Pt nanoparticles supported on Ti_3_C_2_T_x_.^[^ ^45^ ^]^ As shown in Figure S9, Supporting Information, commercial 20 Pt/C exhibits the rapid degradation at overpotential of −10 mA cm^−2^ current density. In comparison, no noticeable current degradation was observed on PtS/Ti_3_C_2_T_x_ at a fixed overpotential corresponding to −10 mA cm^−2^ during a 20 h continuous operation (Figure 4g). After 20 h, no distinct peak shifts were observed in the XPS analysis (Figure S10, Supporting Information). Ti_3_C_2_T_x_ was slightly oxidized to TiO_2_, as shown in Figure S10b, Supporting Information; however, this oxidation was negligible for the HER performance. Moreover, PtS particles are well maintained on the Ti_3_C_2_T_x_ surface without morphology changes, as shown in Figure S11, Supporting Information. Figure 4h and i compare the overpotential and Tafel slope of PtS/Ti_3_C_2_T_x_ with previously reported HER catalysts, and TOF values of previous literature are shown in Table S1, Supporting Information. The PtS/Ti_3_C_2_T_x_ heterostructure demonstrates both the lowest overpotential and the smallest Tafel slope among reported Pt‐ and MXene‐based catalysts, underscoring its potential as a highly active, durable, and Pt‐conserving HER catalyst.^[^ ^45^, ^46^, ^47^, ^48^, ^49^, ^50^, ^51^, ^52^ ^]^ These results highlight the high HER activity of PtS/Ti_3_C_2_T_x_. Since only a very small amount of PtS_2_ is present and it is known to be less active than PtS,^[^ ^31^ ^]^ the catalytic performance can be mainly ascribed to PtS together with Ti_3_C_2_T_x_.

## 3 Conclusion

In summary, we developed a direct synthesis approach to construct PtS‐decorated Ti_3_C_2_T_x_ MXene heterostructures by thermally decomposing a single‐source precursor, Pt(dmampS)_2_, on exfoliated Ti_3_C_2_T_x_ surfaces. This method enables uniform nanoparticle dispersion and strong interfacial coupling without the need for additional surfactants or binders. Structural and chemical characterizations confirmed the successful formation of PtS nanoparticles firmly anchored to the Ti_3_C_2_T_x_ surface. The resulting heterostructure exhibits significantly enhanced HER performance compared to pristine Ti_3_C_2_T_x,_ including a low overpotential (–104 mV vs. RHE), a reduced Tafel slope (48.3 mV dec^−1^), and improved electrochemical stability and charge transfer characteristics. These results clearly demonstrate the potential of precursor‐driven interfacial engineering in activating otherwise inert MXene surfaces, providing a practical and scalable strategy for developing highly efficient, durable, and Pt‐conserving HER catalysts.

## 4 Experimental Section

4.1

4.1.1

##### Synthesis of Single‐Source Precursor Pt(dmampS)_2_

All synthesis processes were carried out under an inert atmosphere, such as oxygen‐free nitrogen, using standard Schlenk techniques or in an argon‐filled glove box. THF was purified with an Innovative Technology PS‐MD‐4 solvent purification system. The synthesis process of Lithium 1‐(dimethylamino)‐2‐methylpropane‐2‐thiolate (Li(dmampS)) was modified from a previous method.^[^ ^53^ ^]^ Pt(dmampS)_2_ was synthesized by mixing PtCl_2_ (1.06 g, 4 mmol) and Li(dmampS) (1.11 g, 8 mmol) in THF(100 mL) and stirring at ambient temperature for 18 h. After the reaction, solid precipitates were removed by filtration, followed by residual solvent evaporation under vacuum to obtain a crude product. Subsequent sublimation at 130 °C under vacuum (0.7 Torr) resulted in a yellow solid pure product (0.9 g, 50%). ^1^H NMR (C_6_D_6_, 400 MHz): *δ* 1.63 (s, 6H), 2.20 (s, 2H), and 2.70 (s, 6H). ^13^C NMR (C_6_D_6_, 100 MHz): *δ* 33.16, 43.07, 57.48, and 87.59. Elemental Anal. Calcd for C_12_H_28_N_2_S_2_Pt: C, 31.36; H, 6.14; N, 6.10; S, and 13.95. Found: C, 31.92; H, 6.15; N, 6.06; and S, 14.01%. EI‐MS: *m/z* = 459 [Pt(dmampS)_2_]^+^ Figure S12, Supporting Information. The synthesizing process of Pt(dmampS)_2_ is shown in Figure 1a.

##### Synthesis of Exfoliated Ti_3_C_2_T_x_ MXene from Ti_3_AlC_2_ MAX

1 g of Ti_3_AlC_2_ powder was added to 10 mL of mixed solution, which contained 3 mL of HF aqueous solution (48%), 6 mL of HCl aqueous solution (35%), and 1 mL deionized (DI)‐water, in a Teflon round bottom flask. The mixture was vigorously stirred at room temperature for 24 h for Al etching. After the etching process, the reacted powder was centrifuged and washed with DI‐water at 11 000 rpm for 4 times until the pH of the solution reached ≈7. For exfoliation of Ti_3_C_2_T_x_ MXene sheets, washed powder was dispersed and reacted with 10 mL 8 M LiCl solution and 50 mL DI‐water for 4 h, stirring at 700 rpm. After 4 h, the exfoliated powder was centrifuged and washed with DI‐water at 11 000 rpm for 2 times. Finally, the collected powder was dispersed in 150 mL isopropyl IPA and centrifuged at 3 000 rpm to obtain a few layers of Ti_3_C_2_T_x_ MXene sheets with a solution density reached ≈2 mg ml^−1^.^[^ ^54^ ^]^

##### Fabrication of PtS/Ti_3_C_2_T_x_ Catalyst

To the fabrication of HER catalyst, 1.72 mg of Pt(dmampS)_2_ was dissolved in MXene solution with the same weight percent, and added to the IPA for the solution reached 2 mL. The 200 μl of prepared solution was coated on the carbon plate (1 × 1 cm^2^) and dried on the 80 °C hot plate. Finally, the coated carbon plate was annealed in a 300 °C tube furnace for 30 min under an Ar atmosphere of 100 sccm, and a HER catalyst was fabricated, denoted as PtS/Ti_3_C_2_T_x_. For comparison, the annealed Ti_3_C_2_T_x_ and PtS were prepared under the same conditions as the PtS/Ti_3_C_2_T_x_, and 20% Pt C^−1^ was prepared under the solution density without an annealing process. The process of synthesizing the HER catalyst is shown in Figure 1b.

##### Electrochemical Analysis

The electrochemical performance of PtS/Ti_3_C_2_T_x_ was investigated in a 0.5 M H_2_SO_4_ electrolyte using a ZIVE MP4 electrochemical workstation. All electrochemical measurements were conducted in a three‐electrode system, PtS/Ti_3_C_2_T_x_ as the working electrode, platinum (Pt) as the counter electrode, and Ag/AgCl in 3.5 M KCl as the reference electrode. For comparison, annealed Ti_3_C_2_T_x_ and PtS were used as the working electrode. LSV was carried out under the potential window between 0 to −0.3 V versus RHE with a scan rate of 5 mV s^−1^. EIS was performed frequency range between 10 kHz and 100 mHz with a 10 mV alternating current (AC) amplitude. During the EIS analysis, the bias potential of −0.2 V versus RHE was applied to activate the HER catalyst. The accelerated degradation test (ADT) was conducted after 1,000 cycles of CV under a potential window of −40 mV to 0 V versus. RHE at a scan rate of 100 mV s^−1^, and LSV analysis was performed under the same conditions. Chronoamperometry was conducted for 20 h with an overpotential of −10 mA cm^−2^ current density. All potentials were converted to the RHE scale using the following equation

$$E_{\text{RHE}}=\;E_{\text{Ag/AgCl in 3}\text{.5M KCl}}+\;0.205\;+\;0.059pH$$

where 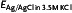 is the measured potential versus the Ag/AgCl (3.5 M KCl) reference electrode, 0.205 V is the standard potential difference between Ag/AgCl (3.5 M KCl) and RHE, and the pH of 0.5 M H_2_SO_4_ electrolyte is considered 0.

The number of active sites of the HER catalyst (N) is calculated from the following equation.

$$N\left(\text{mol}\right)=\frac{Q_{\text{cv}}}{2\times F}=\frac{\int jdV}{2\times \nu \times F}$$

In the above equation, *Q*_cv_ is the amount of charge calculated from CV measurements between 55 and 205 mV versus RHE, which is the electric double layer region. The *j*, *ν*, and *F* are current density (A cm^−2^), scan rate of 50 mV s^−1^, and Faraday constant (96 485 C mol^−1^), respectively. And, the TOF is calculated using the following equation.

$$\text{TOF}=\;\frac{j\times A}{\left(z\times F\times N\right)}$$

Where *j*, *A*, *z*, *F*, and *N* are the current density (A cm^−2^) at the −104 mV versus RHE overpotential, geometric area of working electrode, participating electron during the HER process, Faraday constant (96 485 C mol^−1^), and number of active sites, respectively. In this study, *A* and *z* are 0.5 cm^2^ and 2.^[^ ^55^, ^56^, ^57^, ^58^ ^]^

## Conflict of Interest

The authors declare no conflict of interest.

## Supporting information

Supplementary Material

## Data Availability

The data that support the findings of this study are available from the corresponding author upon reasonable request.
