# Supplementary material for: Direct Growth of Platinum Monosulfide Nanoparticles on MXene via Single‐Source Precursor for Enhanced Hydrogen Evolution Reaction
Source: Small Sci. 2025 Sep 15;5(11):2500407. doi: 10.1002/smsc.202500407 (PMC12622561; doi:10.1002/smsc.202500407)
Supplement: Supplementary file 1 — Supplementary Material [file SMSC-5-2500407-s001.pdf]

Supporting Information

Direct Growth of PtS Nanoparticles on MXene via Single-Source Precursor for Enhanced Hydrogen Evolution Reaction

Younghee Park,[#,a] *Chanwon Park,*[#a,b] *Sunyoung Shin,*[a] *Da Som Song,*[a] *Myung Hyun Kang,*[a] *Chang Gyoun Kim,*[a] *Yun Chan Kang,*[b] *Sung Myung,*[a,*] *and Jongsun Lim*[a,*]

1. Materials

Ti_3AlC_2 MAX powder, hydrofluoric acid (HF, 48 %), sulfuric acid (H_2SO_4, 95.0-98.0 %), 8 M lithium chloride solution (8 M LiCl, 99%), and platinum chloride ($PtCl_2$, 98%), and *n*-butyllithium solution (2.5 M in hexane) were purchased from Sigma-Aldrich. Hydrochloric acid (HCl, 35 %) and isopropyl alcohol (IPA, 99.9%) were bought from Duksan Chemical. Potassium chloride (KCl, 99.0 %) was obtained from Samchun Chemical, and carbon plate (size: 0.20 x 100 x 100 mm) was prepared from Nilaco Corporation. Isobutylene sulfide (98%) was bought from Tokyo Chemical Industry and used as received. 20% Pt/C was purchased from Alfa Aesar.

2. Characterization

^1H and ^{13}C Nuclear magnetic resonance (NMR) were recorded on a Bruker DPX 400 MHz FT-NMR spectrometer with benzene-d^6 as solvent and standard at ambient temperature. Elemental analysis (EA) was performed with a Thermoquest EA-1110 CHNS analyser. Thermogravimetric analyzer (TGA) was performed under atmospheric pressure with N_2 as carrier gas with heating rate of 10°C/min from room temperature to 800°C using a thermogravimetric analyzer (Netzsch, TG 209 F3TG). The crystal structure of MXene was determined by X-ray diffraction (XRD, Rigaku D/Max-2200, Cu Kα) with a scan range of 5 to 80° at a scan rate of 3°/min. The Raman spectrum was performed at a wavelength of 514 nm with a power of 4.3 mW. High electron resolution transition electron microscopy (HR-TEM) and selective area electron diffraction (SAED) were conducted using JEM-2100F HR, JEOL Ltd with 200 kV acceleration voltage. Energy dispersive X-ray spectroscopy was progressed using Tecnai F-30 S-Twin with 300 kV accelartion voltage. The surface composition was investigated to X-ray photoelectron spectroscopy (XPS, K-alpha, Thermo Scientific) using Al-

Kα radiation. The morphology of the PtS/$Ti_3C_2T_x$ before and after chronoamperometry test was examined using a field-emission scanning electron microscope (FE-SEM, Carl Zeiss/Gemeni 560 with an acceleration voltage of 3 kV).

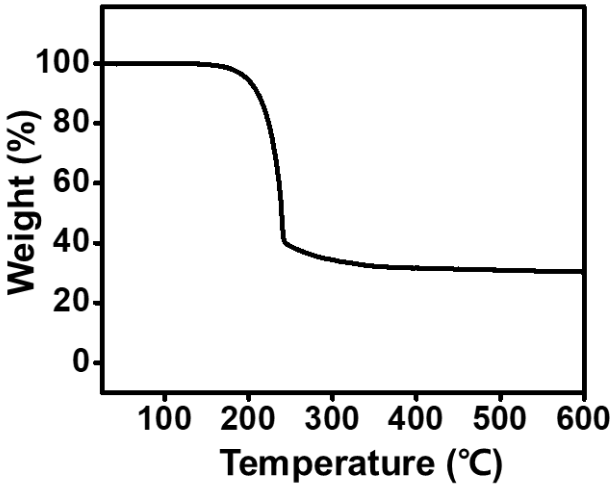

Figure S1. TGA curves of Pt(dmampS)$_2$ to observe thermal decomposition of the precursor.

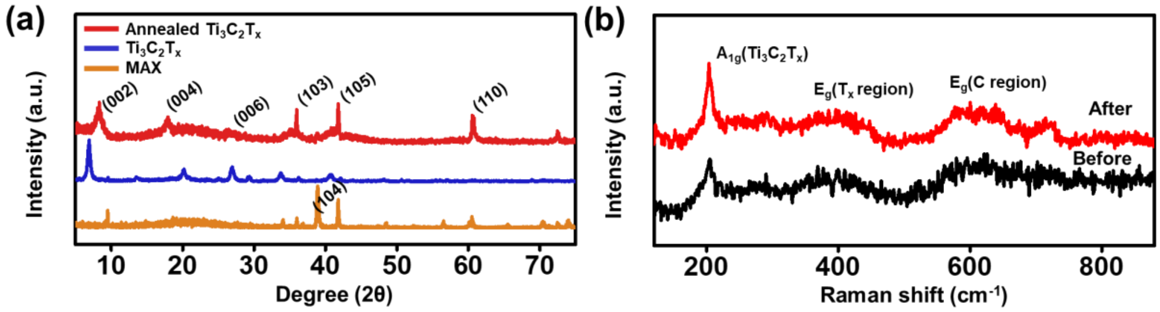

Figure S2. (a) XRD pattern of Ti_3AlC_2 (orange), $Ti_3C_2T_x$ (blue), and PtS/$Ti_3C_2T_x$ (red), (b) Raman spectrum of PtS/$Ti_3C_2T_x$ before annealing (black) and after annealing (red) at 300°C under Ar atmosphere.

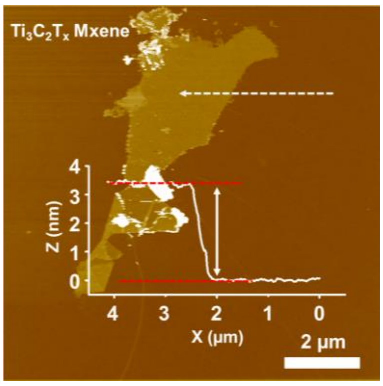

Figure S3. AFM image and height of exfoliated $Ti_3C_2T_x$ MXene.

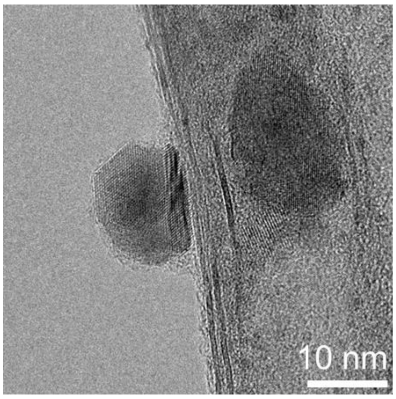

Figure S4. HR-TEM of PtS/$Ti_3C_2T_x$ showed that PtS nanoparticles were well anchored on $Ti_3C_2T_x$ surfaces.

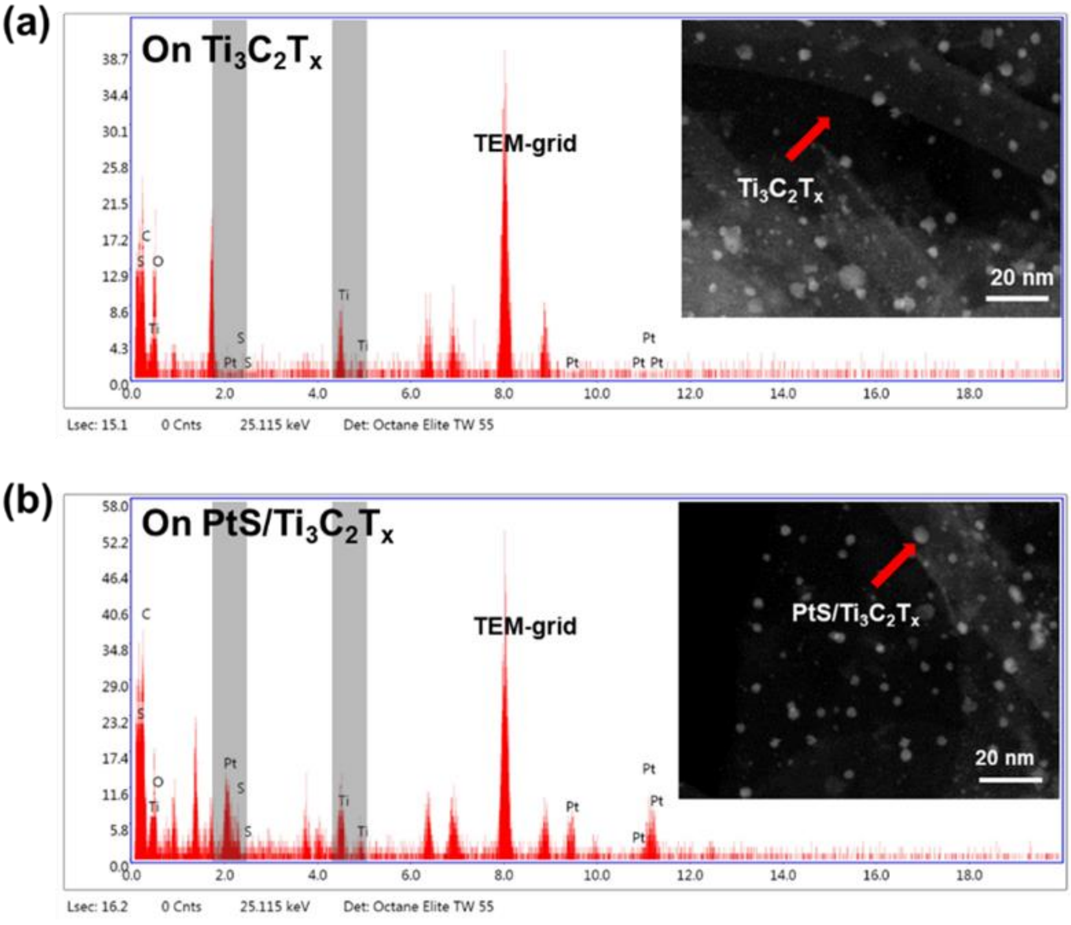

Figure S5. TEM-EDS Spectra and images of PtS/$Ti_3C_2T_x$ collected from different regions of the same sample: (a) the spectrum and image from the $Ti_3C_2T_x$ region and (b) the spectrum and image from the PtS/$Ti_3C_2T_x$ region.

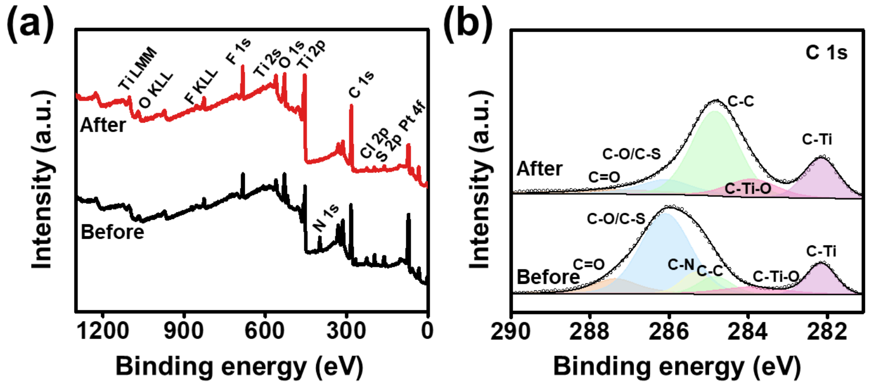

Figure S6 (a) XPS survey spectra of PtS/$Ti_3C_2T_x$ before (black) and after annealing (red), (b) C 1s spectra of before and after annealing PtS/$Ti_3C_2T_x$. The annealing process was carried out at 300°C under the Ar atmosphere.

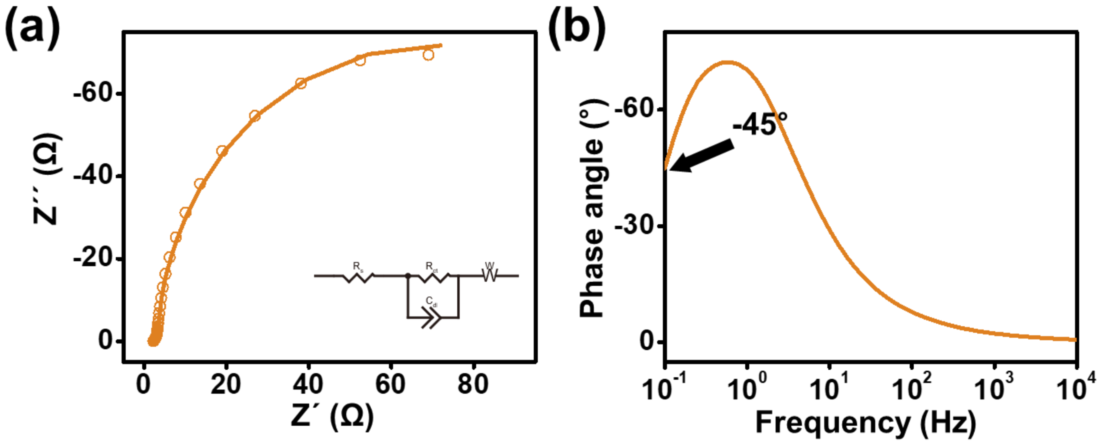

Figure S7. (a) Nyquist plot of $Ti_3C_2T_x$ fitted using equivalent circuit inset figure, (b) Bode plot of $Ti_3C_2T_x$. EIS was conducted with a bias of -0.2 V vs. RHE.

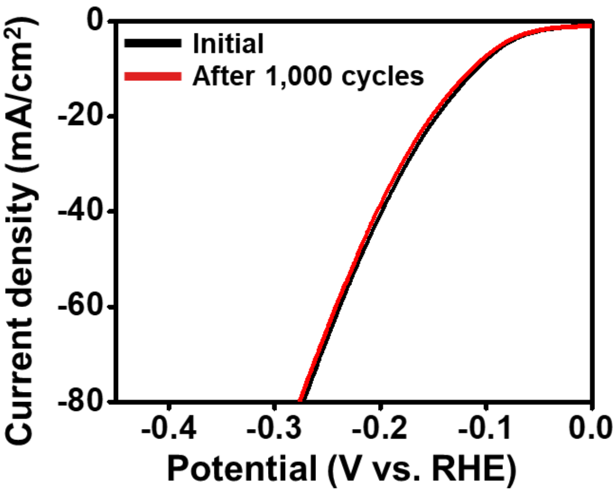

Figure S8. Accelerated degradation test of PtS/$Ti_3C_2T_x$ after CV 1,000 cycles with potential window -40 mV to 0 V vs. RHE at 100 mV/s scan rate.

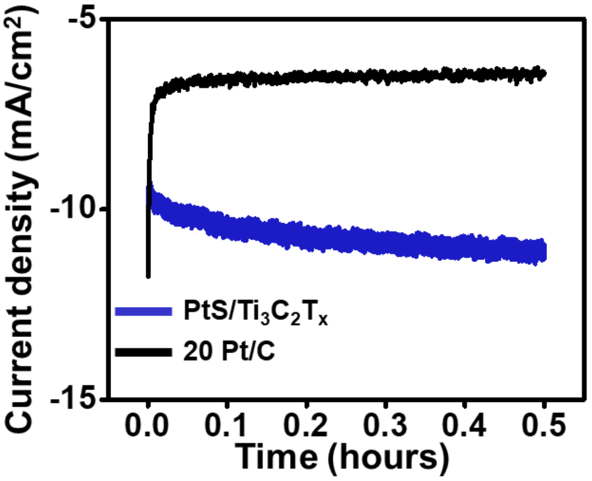

Figure S9. Chronoamperometry measurement of commercial 20 Pt/C (black) and PtS/$Ti_3C_2T_x$ (blue) with each overpotential of -10 mA cm^{-2} current density.

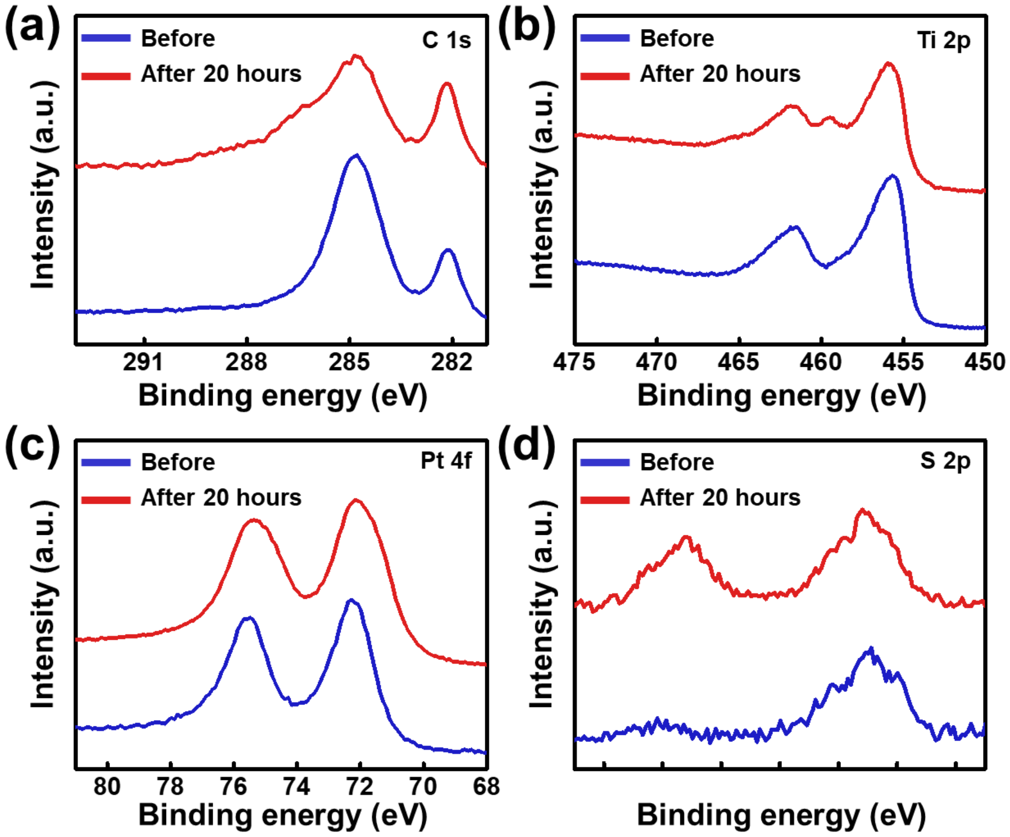

Figure S10. XPS spectra of (a) C 1s, (b) Ti 2p, (c) Pt 4f, and (d) S 2p for PtS/$Ti_3C_2T_x$ before and after chronoamperometry measurement.

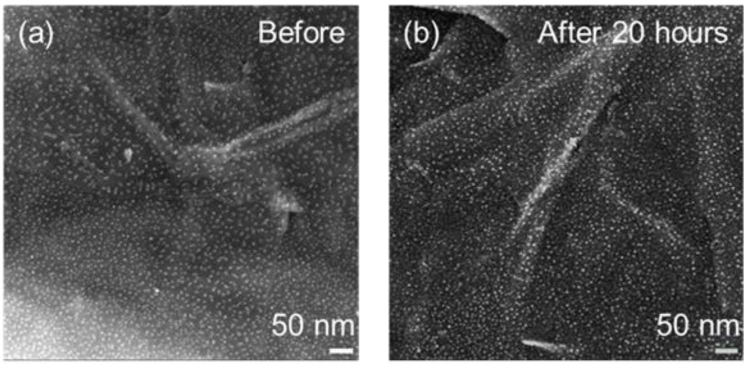

Figure S11. SEM images of PtS/$Ti_3C_2T_x$ recorded (a) before and (b) after 20 hours the CA test, showing that PtS nanoparticles remain uniformly anchored on the $Ti_3C_2T_x$ surface.

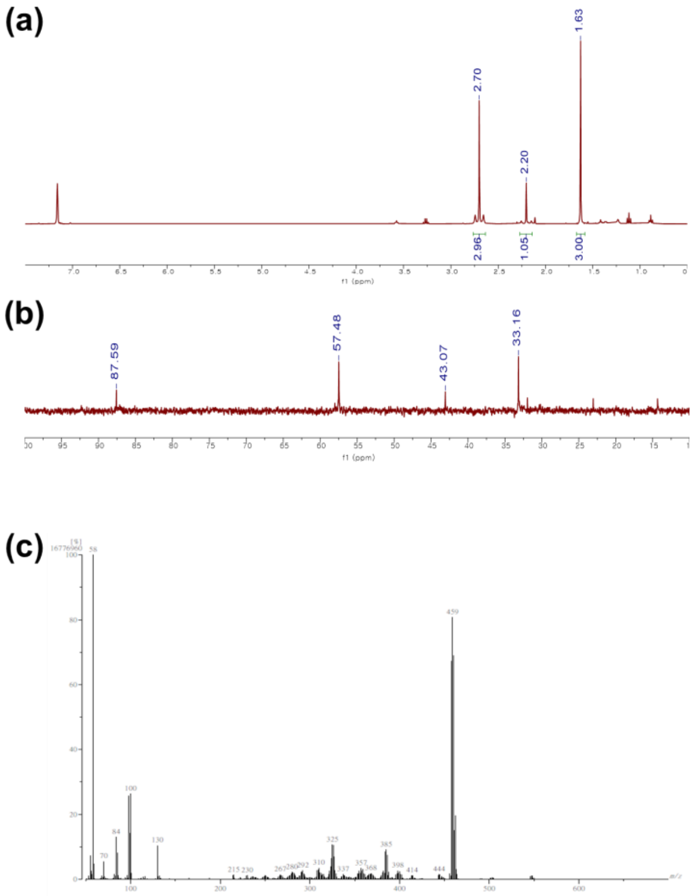

Figure S12. (a) ^{1}H and (b) ^{13}C NMR spectrum of $Pt(dmampS)_2$ in benzene-d6, (c) mass spectrum of $Pt(dmampS)_2$.

Table S1. TOF values of various Pt- and $Ti_3C_2T_x$- based HER catalysts.

	TOF (s^{-1})	Reference
**$Pt/TiO_2/Ti_3C_2$**	2.50	[45]
**CoS_2–P–$Ti_3C_2T_x$**	0.0014	[47]
**Pt/SnO_2@NPC-300**	4.13	[57]
**TiO_2@TiC/Pt-5**	5.15	[58]
**20 Pt/C**	3.50	This work
**PtS/$Ti_3C_2T_x$**	4.43	This work
